# Coronary Artery Spasms Mimicking Acute ST-Elevation Myocardial Infarction in Dengue Haemorrhagic Fever

**DOI:** 10.1155/2020/6310569

**Published:** 2020-02-11

**Authors:** Chathurika L. Dandeniya, Indika B. Gawarammana, Gamini Weerakoon

**Affiliations:** ^1^Department of Medicine, Faculty of Medicine, University of Peradeniya, Peradeniya, Sri Lanka; ^2^Cardiology Unit, Teaching Hospital, Kandy, Sri Lanka

## Abstract

Dengue is an arboviral febrile illness endemic in many tropical and subtropical regions with frequent epidemics. Although most cases are self-limiting, progression into dengue haemorrhagic fever can have dire outcomes. The course can also be complicated by infrequent occurrence of unusual clinical manifestations which are increasingly recognized. We describe the case of a 16-year-old previously healthy girl diagnosed with dengue, who went on to develop severe ischaemic-type central chest pain towards the end of the critical phase of dengue haemorrhagic fever. Urgent investigation revealed acute ST-segment elevations in the high lateral leads of a surface electrocardiogram which completely reverted to normal within 2 hours, associated with elevated cardiac biomarkers but normal findings on transthoracic 2D echocardiography. She was managed with high-dose statins, nitrates, and pain relievers and made an uneventful recovery without any further episodes. The possible explanation for the presentation was focal myocarditis leading to coronary artery spasms.

## 1. Background

Dengue has emerged as a public health threat with abundant distribution in the tropics and subtropics, with an estimated occurrence of 50 million cases around the world annually [1]. The culprit, an arbovirus of flaviviridae family, is transmitted by mosquitoes of the genus Aedes [1]. Endemic regions including Sri Lanka experience frequent seasonal epidemics [2]. In addition to the well-defined complications such as plasma leakage [1], haemorrhage [1] and myocarditis [1, 3], less-frequent associations usually described in case reports or case series can complicate the course of disease. Examples include haemophagocytic lymphohistiocytosis [4] and rare neurological complications like neuromyelitis optica [5]. We describe a case of probable coronary artery spasms complicating a case of dengue haemorrhagic fever (DHF) in an otherwise healthy young patient with no known risk factors for coronary artery disease.

## 2. Case Presentation

A 16-year-old previously healthy girl presented to the emergency department during a dengue outbreak, with a three-day history of fever associated with headache, arthralgia, and myalgia. She was haemodynamically stable without evidence of plasma leakage.

A positive NS1 dengue antigen test confirmed a clinical suspicion of dengue fever, and she was admitted for observation. On the fifth day of illness, she demonstrated evidence of plasma leakage in the form of mild ascites and a right-sided pleural effusion, which was confirmed on ultrasonography. She was managed as being in the critical phase of DHF. Over the next 48 hours, she was monitored closely and was managed according to the national guidelines on dengue.

Following initial steady improvement, she suddenly deteriorated towards the end of the critical phase (46 hours since the presumed onset of leaking) with acute onset of severe tightening central chest pain radiating to the left upper limb and jaw associated with nausea and sweating. Pain lasted for more than 20 minutes, finally subsiding with morphine and sublingual nitrates. Her pulse rate and blood pressure remained stable.

## 3. Investigations

An urgent electrocardiogram (ECG) demonstrated ST-segment elevation in lead 1 and aVL. A high-sensitivity cardiac troponin I titre 2 hours after the onset of the chest pain was 1.42 *µ*g/l (normal level <0.01). An urgent 2D echocardiogram failed to reveal any regional wall motion abnormalities or structural cardiac defects, and the cardiac systolic function was preserved. Coronary angiography was precluded by the severe thrombocytopaenia (28 × 10^9^/l) related to dengue and the critical stage of DHF. Cardiac magnetic resonance imaging (MRI) was considered but could not be performed due to nonavailability of facilities. Her symptoms resolved over the next 10–15 min with treatment using sublingual nitrates. Interestingly, the ECG was repeated 2 hours from the onset of the chest pain, and subsequent serial ECGs showed complete resolution of the high lateral ST-segment elevations.

The course of the parameters in a complete blood count and alanine aminotransferase level is shown in Table 1.

## 4. Treatment

Under supervision of the cardiologist, she was given 40 mg of atorvastatin. Antiplatelet agents, thrombolysis, or urgent angiography with percutaneous coronary intervention were deferred due to severe thrombocytopaenia (28 × 10^9^/l) and the critical stage of DHF. Considering the transient nature of the incident, ST-segment elevation on ECG with rapid complete resolution, grossly elevated specific cardiac biomarkers, and the absence of any regional abnormalities on 2D echocardiogram, transient coronary artery spasm probably due to focal myocarditis was considered to have caused the clinical picture. This was also supported by the complete resolution of her symptoms within minutes of administering sublingual glyceryl trinitrate: a vasodilator. Over the next 24 hours, she was closely monitored in the intensive care unit. There were no further similar episodes, and she made a complete recovery. She was discharged on the 11^th^ day of the illness without further sequelae and has remained well on follow-up.

## 5. Discussion

Cardiac involvement in dengue fever is a previously identified phenomenon [3]. This can be in various forms including acute myocarditis, acute pericarditis, cardiac failure, asymptomatic elevation of cardiac biomarkers, and arrhythmias [3], among others.

Following a thorough literature search through PubMed and MEDLINE, only a few case reports were found where the clinical presentation had suggested acute myocardial infarction associated with dengue fever [6–9]. Further evaluation in each of these cases revealed viral myocarditis rather than myocardial infarction to be the actual pathology. But on studying these case records, in addition to the acute severe chest pain, ischaemic-type ECG changes, and the elevated cardiac biomarkers, 2D echocardiography had demonstrated globally impaired left ventricular function and a reduced ejection fraction supportive of myocarditis. Findings on serial ECGs were not provided in the description.

The described case demonstrates some unique features. The ECG changes, rather than being generalized as in most cases of myocarditis, were strictly confined to a small limited region (high lateral). They were rapidly transient, with the ST-segment elevations returning to baseline within 2 hours. Although the cardiac biomarkers were raised, 2D echocardiography performed by an experienced cardiologist failed to demonstrate any global or regional wall motion abnormalities or any impairment in left ventricular function. In addition, on further close observation, the patient did not develop any further episodes of chest pain or shortness of breath and remained symptom-free without any specific medication. All these features support the conclusion that her symptoms were produced by a transient phenomenon with no permanent residual damage in a background of DHF. Although coronary angiography was precluded by thrombocytopaenia and the possible procedure-related risks posed by DHF, it appears safe to assume that the cause of this episode was a transient coronary artery spasm. A coronary angiogram upon recovery was refused by the parents of the patient. The exact reason for this clinical phenomenon is not clear but focal myocarditis is a possible cause. The gold standard in diagnosing this is endomyocardial biopsy, but invasive nature of this makes the second line of cardiac MRI a more attractive option [10]. Neither of these could be performed in the index case.

Dengue serovar typing was not done in this case. However, it is interesting to note that serotypes DEN 1, 2, and 3 have been reported to cause myocarditis in the literature, whereas no reports of DEN 4 causing this clinical manifestation exist [9, 11]. Similarly, no literature could be found to indicate whether primary dengue fever behaves in any way statistically significantly different to secondary dengue fever (reinfection) when it comes to myocarditis. Both these factors might have potentially contributed to this unusual clinical presentation and may be areas for future research.

Although focal myocarditis following viral infection has rarely been reported previously [10], coronary artery spasm or focal myocarditis has never been reported in the context of underlying dengue infection in the published medical literature.

## 6. Conclusion

Reports of unusual manifestations of dengue fever keep accumulating in the medical literature. This case calls to attention the possibility of coronary artery spasm as a differential diagnosis of acute ischaemic-type chest pain in the patient with dengue infection.

## Figures and Tables

**Table 1:** Course of the parameters in a complete blood count and alanine aminotransferase level.

Day of illness	WBC (×10^9^/l)	Plt (×10^9^/l)	PCV (%)	Hb (g/dl)	ALT (U/l)
3	6.3	175	37.6	11.5	
4	5.0	142	35	11.3	
5 (start of critical phase)	3.5	79	36	11.7	132
6	4.2	28	38	12.2	
7	6.7	33	35	11.6	148

WBC: white blood cell count; Plt: platelet count; PCV: packed cell volume; Hb: haemoglobin; ALT: alanine aminotransferase.
